# Proton Density Fat Fraction Spine MRI for Differentiation of Erosive Vertebral Endplate Degeneration and Infectious Spondylitis

**DOI:** 10.3390/diagnostics12010078

**Published:** 2021-12-30

**Authors:** Frederic Carsten Schmeel, Asadeh Lakghomi, Nils Christian Lehnen, Robert Haase, Mohammed Banat, Johannes Wach, Nikolaus Handke, Hartmut Vatter, Alexander Radbruch, Ulrike Attenberger, Julian Alexander Luetkens

**Affiliations:** 1 Department of Neuroradiology, University Hospital Bonn, Rheinische Friedrich-Wilhelms-Universität Bonn, 53127 Bonn, Germany; asadeh.lakghomi@ukbonn.de (A.L.); nils.lehnen@ukbonn.de (N.C.L.); robert.haase@ukbonn.de (R.H.); alexander.radbruch@ukbonn.de (A.R.); 2 Department of Neurosurgery, University Hospital Bonn, Rheinische Friedrich-Wilhelms-Universität Bonn, 53127 Bonn, Germany; mohammed.banat@ukbonn.de (M.B.); johannes.wach@ukbonn.de (J.W.); hartmut.vatter@ukbonn.de (H.V.); 3 Department of Diagnostic and Interventional Radiology, University Hospital Bonn, Rheinische Friedrich-Wilhelms-Universität Bonn, 53127 Bonn, Germany; nikolaus.handke@ukbonn.de (N.H.); ulrike.attenberger@ukbonn.de (U.A.); julian.luetkens@ukbonn.de (J.A.L.)

**Keywords:** spondylitis, osteochondrosis, intervertebral disc degeneration, fat fraction, quantitative imaging

## Abstract

Vertebral Modic type 1 (MT1) degeneration may mimic infectious disease on conventional spine magnetic resonance imaging (MRI), potentially leading to additional costly and invasive investigations. This study evaluated the diagnostic performance of the proton density fat fraction (PDFF) for distinguishing MT1 degenerative endplate changes from infectious spondylitis. A total of 31 and 22 patients with equivocal diagnosis of MT1 degeneration and infectious spondylitis, respectively, were retrospectively enrolled in this IRB-approved retrospective study and examined with a chemical-shift encoding (CSE)-based water-fat 3D six-echo modified Dixon sequence in addition to routine clinical spine MRI. Diagnostic reference standard was established according to histopathology or clinical and imaging follow-up. Intravertebral PDFF [%] and PDFFratio (i.e., vertebral endplate PDFF/normal vertebrae PDFF) were calculated voxel-wise within the single most prominent edematous bone marrow lesion per patient and examined for differences between MT1 degeneration and infectious spondylitis. Mean PDFF and PDFFratio of infectious spondylitis were significantly lower compared to MT1 degenerative changes (mean PDFF, 4.28 ± 3.12% vs. 35.29 ± 17.15% [*p* < 0.001]; PDFFratio, 0.09 ± 0.06 vs. 0.67 ± 0.37 [*p* < 0.001]). The areas under the curve (AUC) and diagnostic accuracies were 0.977 (*p* < 0.001) and 98.1% (cut-off at 12.9%) for PDFF and 0.971 (*p* < 0.001) and 98.1% (cut-off at 0.27) for PDFFratio. Our data suggest that quantitative evaluation of vertebral PDFF can provide a high diagnostic accuracy for differentiating erosive MT1 endplate changes from infectious spondylitis.

## 1. Introduction

Erosive osteochondrosis (EO) of the spine constitutes a special form of disc degeneration in which loss of water in the nucleus pulposus leads to a loss of resilience in the fibers of the annulus fibrosus, resulting in increased mobility of the affected disc segment. The segmental instability may cause an inflammatory reaction in the adjacent bone marrow compartments which evolves in different stages and is commonly referred to as “Modic changes” [1]. Despite the common terminology of endplate-associated findings, these are in fact signal variations that can also extend into the vertebral body to varying degrees [2]. In Modic type 1 (MT1) stage, thought to indicate an ongoing inflammatory process in EO, vertebral bone marrow compartments close to the intervertebral discs are histopathologically replaced by fibrovascular tissue, accompanied by circumscribed edema zones which might correspond to endplate microfractures [3]. Recent research additionally suggests a multifactorial genesis in which activated degeneration is maintained by a proinflammatory milieu of the intervertebral disc, thereby favoring low-grade bony infections [4]. Assessment of EO is an important clinical task because of the positive and very specific association between magnetic resonance imaging (MRI) evidence of MT1 changes and non-specific lower back pain [5].

In the setting of acute non-specific back pain, however, infectious spondylitis is an important differential diagnosis, which in early stages may also manifest with circumscribed areas of edema near the vertebral endplates on conventional morphologic MRI [6]. Conventional MRI may provide important information on signal abnormalities of bone marrow and adjacent soft tissue which can help to diagnose the underlying pathology. However, despite a variety of morphological criteria that may aid differentiating EO from spondylitis [7], conventional MRI sequences may lack specificity, especially in early stage spondylitis without endplate destruction, height decrease or peri-vertebral abscess formation. While clinical and laboratory signs of inflammation are usually inconspicuous in patients with MT1 degeneration, laboratory changes may also be absent in patients with spondylitis, especially in the elderly [8]. Thus, establishing a differential diagnosis between EO and spondylitis can become a diagnostic challenge, as these two entities may mimic each other in terms of both clinical and conventional MRI findings. In rare cases, additional costly and potentially harmful invasive investigations might be required to confirm the nature of the endplate-associated edema. 

It has been proposed that potentially more accurate quantitative MR imaging protocols would improve investigations of the etiology and clinical significance of endplate-associated vertebral edema. Initially aimed to investigate body composition and hepatic steatosis, quantitative chemical-shift encoding complex-based water-fat MRI has emerged as a quantitative imaging biomarker for diagnosis and monitoring of various bone marrow pathologies, including osteoporosis [9,10], intervertebral disk degeneration [11], ankylosing spondylitis [12], focal vertebral lesions, and hematological neoplasms [13,14]. This quantitative MRI technique enables the spatially resolved assessment of the proton density fat fraction (PDFF) in vertebral bone marrow and has been validated against both the histopathologically determined fat content of human bone samples [15] and the MR-spectroscopy based in vivo fat fraction estimations of spine marrow [16]. However, there is as yet no existing study on the use of quantitative PDFF in vertebral bone marrow in order to assess the nature of different types of vertebral endplate-associated edema.

Therefore, the purpose of this inter-individual diagnostic study was to evaluate the discriminatory value of the PDFF derived from CSE-based water-fat MRI in patients with MT1 resembling bone marrow changes to confirm the presence of true degenerative endplate changes and to reduce concerns about possible spondylitis. 

## 2. Materials and Methods

All procedures performed in studies involving human participants were in accordance with the ethical standards of the institutional and/or national research committee and with the 1964 Helsinki Declaration and its later amendments or comparable ethical standards. Institutional review board approval for this retrospective study (approval no. 65/21, Medical Faculty, University of Bonn) has been obtained prior to evaluation and written informed patient consent was waived.

### 2.1. Study Population

Adult patients (>18 years of age) referred for spine MRI because of non-specific back pain or clinical suspicion of spondylodiscitis between April 2015 and December 2020 were retrospectively reviewed to select a total of 53 patients (30 men; mean age 65 years, range 19–97 years) with MR imaging features resembling MT1 degenerative changes as determined on morphologic T1-weighted and (fat-suppressed) T2-weighted MR images with mono- or multisegmental involvement according to the classification established by Modic et al. [1,17]. For this purpose, a board-certified investigator with more than 10 years’ experience in interpreting MR imaging studies of the spine (A.L.) screened eligible MRI scans for characteristic MT1 and spondylitis suspicious changes and decided on the inclusion of patients into this study after reviewing the initial spine MRI. Eligible patients had undergone a standardized CSE-based water-fat MRI with a 3D spoiled six-echo modified Dixon gradient-echo sequence (mDIXON Quant, Philips Healthcare, Best, The Netherlands) in addition to routine clinical spine MRI and were followed up clinically for at least 6 months. Exclusion criteria included the presence of an acute vertebral fracture or intervertebral disc herniation, disseminated, or diffuse bone marrow disease such as metastatic condition or hematologic disorder, documentation of recent or concomitant antibiotic/anti-inflammatory therapy, previous or concurrent chemotherapy (including angiogenesis inhibitors) and/or radiotherapy, bisphosphonate and/or growth colony-stimulating factor treatment, and previous surgery or metallic implants in the spine segment under investigation.

### 2.2. Diagnostic Standard of Reference

A total of 53 patients were identified, 31 with MT1 degenerative changes and 22 with infectious spondylitis.

The diagnosis of infectious spondylitis (group 1) was established with confirmatory biopsy during operative restoration and/or spinal instrumentation in 16/22 patients and on the basis of clinical, laboratory and imaging follow-up in 6/22 patients who were treated conservatively. Biopsy-positive cases in 16/22 patients proved spondylitis with Staphylococcus aureus in 8 patients, Staphylococcus epidermidis in 1 patient, Enterobacter species in 1 patient, Enterococcus faecalis in 2 patients, Cutibacterium acnes in 1 patient, Mycobacterium avium in 2 patients, and Mycobacterium tuberculosis in 1 patient. In the 6/22 patients without available biopsy, suspicious clinical findings suggestive of spondylitis were back pain on heel strike, impaction and percussion, pain on inclination or reclincation and back pain worsening at night, either with or without neurological deficits. Suspicious laboratory findings in these patients included an elevated erythrocyte sedimentation rate, elevated levels of C-reactive protein and leukocytosis. Positive blood culture results in these 6/22 patients were considered as supporting evidence of infection and revealed Staphylococcus aureus in 3 patients, Staphylococcus epidermidis in 1 patient and Escherichia coli in 2 patients. MR imaging follow-up after 2–7 months in 3 of these patients confirmed partial resolution of edematous endplate changes after conservative therapy.

There was no imaging or clinical suspicion of infection in 27/31 patients with MT1 degenerative changes (group 2). All of these patients were followed up clinically, with 6 of whom undergoing at least one follow-up MRI after >5 months, which ultimately ruled out the differential diagnosis of spondylitis in favor of degenerative MT1 erosion, either because of resolution of clinical symptoms or by follow-up MRI showing resolution of edema in the previously suspicious areas or transformation to MT2 endplate changes. A total of 4/31 patients with MT1 degenerative changes had MR imaging signal changes beyond typical endplate-associated edema (group 3), so that the interpreting radiologist could not exclude an underlying infection on an imaging basis only (either because of diffuse hyperintense T2 signal of the affected vertebrae or high T2 disk signal and disk enhancement). These 4/31 patients had no clinical suspicion of infection, including lack of any laboratory data to support infection and negative blood cultures. Additional MR imaging during a 1–6 months follow-up period in 3/4 of these patients revealed either no changes or partial resolution of edematous endplate changes, and follow-up with computed tomography (CT) after 2 weeks and 5 months in 1/4 of these patients showed no cortical or trabecular destruction in the affected vertebrae. While infection could not be definitely excluded on an imaging basis, there was no specific anti-infective treatment, and the presumed resolution of symptoms during clinical follow-up made infection highly unlikely. The corresponding 4 lesions in these 4/31 patients were therefore deemed as MT1 changes on a clinical basis. For comparative purposes, these 4/31 patients were added to a dedicated subgroup 3 to allow further comparisons between patients with and without discrepancy in clinical and radiologic reporting. 

### 2.3. MR Imaging

MR imaging was performed on clinical 1.5T and 3.0T systems (Ingenia, Philips Healthcare, Best, The Netherlands). Morphological MR imaging of the spine was acquired according to the routine clinical MRI protocol used at our institution which included at least a sagittal T1-weighted spin-echo (450–750/6–12 [repetition time (TR) msec/echo time (TE) msec]) and T2-weighted turbo spin-echo sequence (3000–5000/80–120 [TR/TE]) as well as a sagittal T2 spectral attenuated inversion recovery (SPAIR)-weighted turbo spin-echo sequence (3000–5000/50–120 [TR/TE]). In patients with suspected spondylitis, morphological imaging included an additional contrast-enhanced T1-weighted spin-echo sequence performed in sagittal orientation after i.v. administration of Gd-DO3A-butrol (Gadovist). Field of view, matrix size, slice thickness, and interslice gap were slightly different among anatomic regions and scanners.

To determine the relative fat fraction fat/(water + fat), i.e., the PDFF, a 3D spoiled gradient-echo modified Dixon sequence (mDixon Quant, Philips Healthcare, Best, The Netherlands) was acquired in sagittal orientation. This sequence acquires six evenly spaced echoes to account and correct for T2* effects in PDFF estimation [18], uses a low flip angle of 3° to limit T1-bias [19], and uses a multi-peak fat modelling by incorporating a pre-calibrated seven-peak fat spectrum in the signal model as proposed by Yu et al. [20]. The sequence parameters are as follows: TR/TE1 = 8/1.15 ms; ΔTE: 1.15 ms; averages = 1; acquisition matrix = 175 mm × 100 mm; field-of-view (FH × AP × RL) = 350 mm × 200 mm × 160 mm; SENSE-factor = 2; scan time = 0:37 min. The parametric PDFF map was automatically calculated by the imager software (Ingenia vendor software v5.1 or above).

### 2.4. Image Analysis

Image analyses were performed by a board-certified radiologist with 8-years’ experience in interpreting spine MRI (F.C.S.), blinded to patient-related information. In each patient, the single most prominent lesion (i.e., the lesion with largest possible diameter) was defined based on morphologic MRI findings. Morphological imaging sequences and PDFF maps were cross-linked to ensure correct lesion detection and delineation. Free-hand regions of interest (ROIs) were placed at a single slice with the largest possible lesion diameter with each ROI being adapted to the area of hypointense bone marrow signal on the T1-weighted image. Areas close to the rim and adjacent vertebral structures were excluded from the analysis. ROIs were afterwards copied onto the corresponding PDFF map and the mean PDFF [%] values inside each ROI and the corresponding ROI size were recorded. In addition, the mean percentage PDFF of adjacent unaffected vertebrae were determined using circular ROIs, as large as possible, on midline sagittal images. The ratio between the PDFF of suspicious edema zones and normal vertebrae (i.e., endplate change PDFF/normal vertebrae PDFF) was calculated and referred to as PDFFratio.

### 2.5. Statistical Analysis

Statistical analysis was conducted using SPSS (v25.0 and above, IBM, Armonk, NY, USA). Mean ± standard deviation was calculated for all applicable clinical and imaging data, unless otherwise specified. Statistical significance level was set at *p* < 0.05 and tested for independent samples using Mann–Whitney-U-Test (2 groups comparisons for MT1 and infectious lesions, MT1 and normal bone marrow, and for spondylitis and normal bone marrow), chi-square test (cross-tables), or Kruskal–Wallis test with post-hoc testing according to Dunn–Bonferroni (3 groups comparisons). In order to differentiate MT1 and infectious endplate changes, receiver operating characteristic (ROC) analysis was performed and optimal diagnostic cut-off points were selected. Sensitivity, specificity, diagnostic accuracy, positive predictive value (PPV), and negative predictive value (NPV) were additionally calculated.

## 3. Results

The infectious spondylitis group (group 1) comprised of 22 patients (16 men; mean age, 62 ± 22 years, range 19–97 years), the MT1 group (group 2) comprised of 27 patients (12 men; mean age, 68 ± 15 years, range 30–95 years), and the MT1 group with imaging suspicion of spondylitis later disproved clinically (group 3) comprised of 4 patients (3 men; mean age 67 ± 11 years, range 54–80 years). There was no statistically significant difference across the three groups regarding age (*p* = 0.726) and gender (*p* = 0.059) distribution. Twenty-five patients with 13 MT1 degenerative changes and 12 infectious lesions were scanned at 1.5T, whereas 28 patients with 18 MT1 degenerative lesions and 10 infectious spondylitis were examined at 3T. 

On routine clinical spine MRI, all 53 vertebral lesions in the three subgroups were hypointense on T1-weighted and hyperintense on (fat-suppressed) T2-weighted MR images as compared with adjacent healthy bone marrow. Overall, 3 lesions were located in the cervical spine, 5 were located in the thoracic spine, and 45 were located in the lumbar spine. In group 1, 2 lesions were located cervical, 5 thoracic, and 15 lumbar. In group 2, 27 lesions were located in the lumbar spine. In group 3, 1 lesion was located cervical and 3 lesions were located in the lumbar spine.

### 3.1. Quantitative Analysis

PDFF readout was performed in a total of 53 MT1 resembling lesions (i.e., 31 MT1 degenerative changes and 22 infectious spondylitis) and 53 adjacent, morphologically normal appearing vertebral bodies within the spine segment under investigation. Mean ROI size was 222 ± 141 mm^2^. The ROI size in MT1 degenerative changes and infectious spondylitis amounted to 178 ± 109 (range, 59–510) mm^2^ and 283 ± 159 (range, 70–829) mm^2^, respectively, the difference in ROI size between degenerative and infectious lesions being statistically significant (*p* = 0.003).

Quantitative PDFF and PDFFratio values of MT1 degenerative changes and infectious spondylitis showed statistically significant differences as summarized in detail in Table 1. 

**Figure 1:**
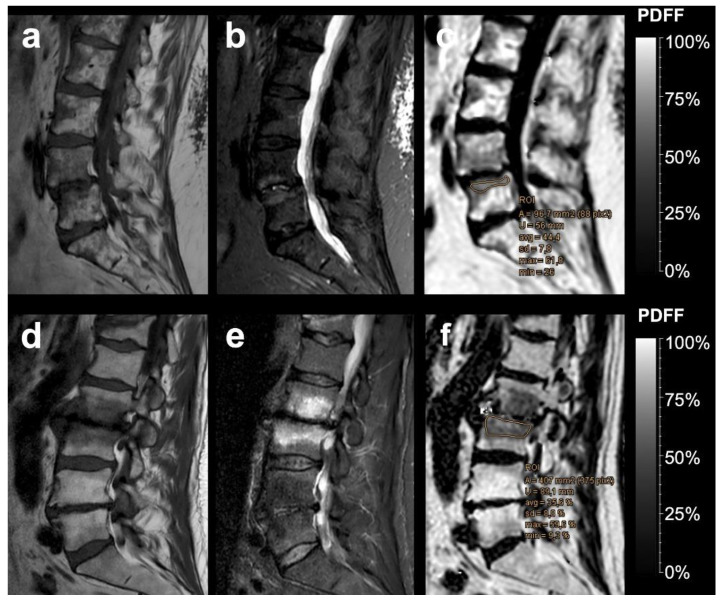
Two examples of vertebral Modic type 1 degenerative endplate changes. Sagittal T1-weighted SE images (**a**,**d**), sagittal T2 SPAIR images (**b**,**e**), and the corresponding PDFF parameter maps (**c**,**f**) with a %-value scale. Using a critical cut-off value of ≤12.9 PDFF% for infectious spondylitis, quantitative water-fat MRI correctly identified the degenerative pathophysiology of both Modic type 1 lesions, as illustrated with exemplary region-of-interest measurements.

**Figure 2:**
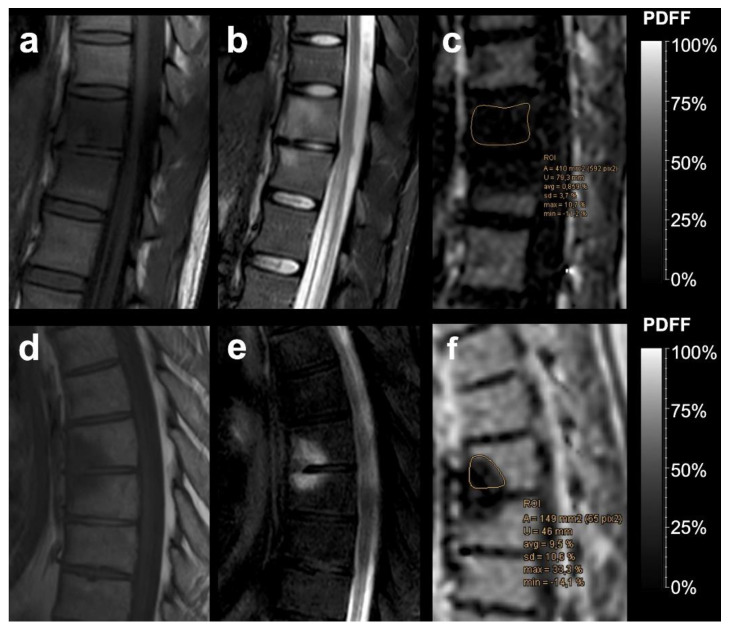
Two examples of histopathological confirmed infectious spondylitis. Sagittal T1-weighted SE images (**a**,**d**), sagittal T2 SPAIR images (**b**,**e**), and the corresponding PDFF parameter maps with a %-value scale (**c**,**f**). PDFF correctly identified infectious lesions at a critical cut-off value of ≤12.9 PDFF% for infectious spondylitis, as illustrated with exemplary region-of-interest measurements. Spondylitis tends to show lower PDFF values than Modic type 1 degeneration.

The mean PDFF was significantly higher in MT1 degenerative changes than in cases with underlying infectious spondylitis: mean PDFF for MT1 degenerative changes (Figure 1) was 35.29 ± 17.15% and mean PDFF for infectious spondylitis (Figure 2) was 4.28 ± 3.12% (*p* < 0.001). Mean PDFFratio for MT1 was 0.67 ± 0.37 and mean PDFFratio for spondylitis was 0.09 ± 0.06 and also significantly different between the two groups (*p* < 0.001). Mean PDFF for normal vertebral bodies was 51.56 ± 17.26% and differed significantly from mean PDFF values for both MT1 (*p* = 0.038) and infectious lesions (*p* < 0.001).

**Figure 3:**
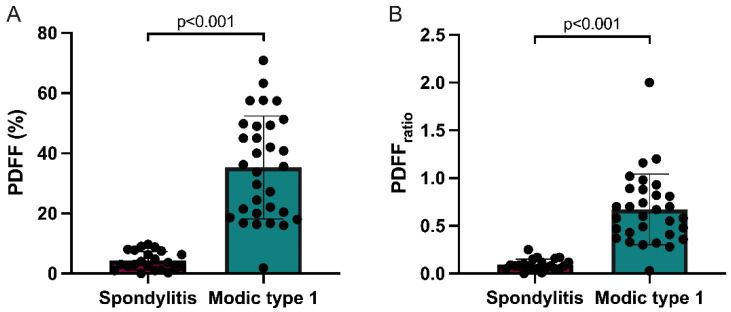
Column charts with individual plotted values show distribution of PDFF (**A**) and PDFFratio (**B**) in infectious spondylitis and Modic type 1 degeneration. Data are presented as mean with standard deviation error bars.

Subclass mean PDFF and PDFFratio values are graphically illustrated in Figure 3. Post-hoc comparisons of the quantitative MR imaging parameters PDFF and PDFFratio across the three patient subgroups are additionally summarized in detail in Table 2 and Table 3. 

Dedicated field-strength specific comparisons between measurements at the 1.5T and 3T scanners showed no statistically significant differences in quantitative PDFF values with respect to MT1 lesions (33.76 ± 18.62% at 1.5T vs. 36.39 ± 16.27% at 3T; *p* = 0.737), infectious spondylitis (3.91 ± 3.47% at 1.5T vs. 4.72 ± 2.74% at 3T; *p* = 0.314), and adjacent healthy bone marrow (46.63 ± 18.61% at 1.5T vs. 55.96 ± 14.65% at 3T; 0.117). Likewise, there was no statistically significant difference in PDFFratio between measurements at 1.5T and 3T regarding MT1 lesions (0.65 at 1.5T vs. 0.68 at 3T; *p* = 0.984) and infectious spondylitis (0.08 at 1.5T vs. 0.1 at 3T; *p* = 0.539).

### 3.2. Diagnostic Performance

Results of ROC analysis are given in Table 4. The AUC for PDFF and PDFFratio was 0.977 (95%CI, 0.931–1; *p* < 0.001) and 0.971 (95%CI, 0.914–1; *p* < 0.001), respectively. Screening all available PDFF and PDFFratio cut-off values for diagnostic performance on ROC analysis revealed that a cut-off value of ≤12.9% for PDFF and ≤0.27 for PDFFratio was best suited to differentiate MT1 degenerative changes from infectious spondylitis, whereby 98% and 98% were correctly identified as MT1 degenerative changes and infectious spondylitis. 

**Figure 4:**
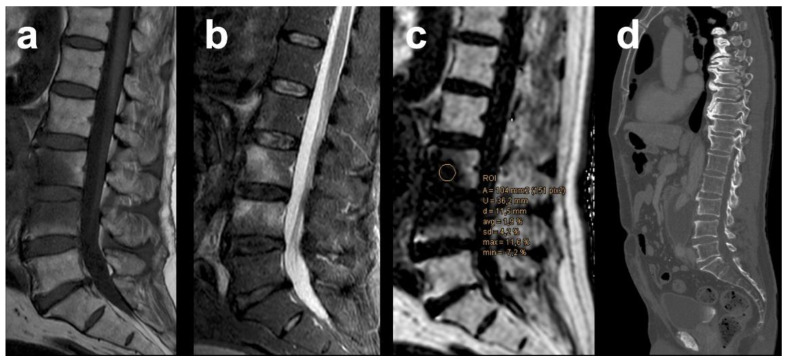
Example of a Modic type 1 degenerative lesion at the lumbar level in a patient with inflammatory infrarenal aortic aneurysm, falsely rated positive as infection by quantitative chemical-shift encoding based water-fat MRI at a critical cut-off value of ≤12.9 PDFF%, probably due to extensive amount of edema. Sagittal T1-weighted SE image (**a**), sagittal T2 SPAIR image (**b**), the corresponding PDFF parameter map (**c**), and follow-up computed-tomography 5 months after aorto-biiliac bypass showing no cortical or trabecular vertebral destruction (**d**).

With a PDFF cut-off value of ≤12.9% for infectious spondylitis, 22/22 lesions were correctly identified as infectious and 30/31 lesions were correctly scored as degenerative, whereas 1 MT1 lesion was falsely rated positive for infection (Figure 4). This yielded a PPV of 95.7%, a NPV of 100%, and an accuracy of 98.1% in distinguishing infectious from MT1 lesions. The PDFFratio also had high diagnostic performance at a cut-off value of ≤0.27 for infection: with 22/22 infectious and 30/31 MT1 degenerative lesions being correctly classified and 1 false-positive rating of a MT1 degenerative lesion, this resulted in a PPV of 95.7%, a NPV of 100%, and an accuracy of 98.1%.

## 4. Discussion

This study evaluated the diagnostic performance of PDFF derived from CSE-based water-fat spine MRI in order to differentiate MT1 vertebral endplate changes from infectious spondylitis, all of whom presenting with bone marrow edema on conventional MRI. The major finding revealed that in a population with a relatively wide age range, there are statistically significant differences in both PDFF and normalized PDFFratio values between MT1 and infectious spondylitis. By using a PDFF cut-off value of ≤12.9% for spondylitis, ROC curves yielded a sensitivity of 100% and a specificity of 97% with a corresponding AUC of 98% in the differentiation of MT1 from infection. These results were accomplished with an excellent accuracy of 98%, a PPV of 96%, and a NPV of 100%. PDFFratio also allowed for a highly accurate differentiation between both entities, albeit minimally weaker than the absolute PDFF with respect to the corresponding ROC curves. Thus, quantitative water-fat spine MRI can identify spondylitis with a fairly high diagnostic accuracy on the basis of a reduction in absolute PDFF and normalized PDFFratio, while adding just a minute of additional acquisition time to the whole examination.

In the setting of infection, there is displacement and depletion of fatty bone marrow due to inflammation-induced fluid extravasation, resulting in hypointense signal on T1-weighted images and hyperintense signal on T2-weighted MR images [7]. Because of the similarity between erosive MT1 degeneration and infection on conventional MRI, the differential diagnosis requires the use of more sensitive methods that can aid in the distinction, especially in the setting of early stage infection in the absence of distinct characteristics such as vertebral body endplate destruction or abscess formation. Traditionally, qualitative and semiquantitative methods have been employed to assess the likelihood of spondylitis on conventional MRI. Older studies either measured the percentage of edema area within affected vertebral bodies [21] or assessed the shape and separability of the morphologic edema pattern within affected vertebrae [22] as diagnostic criteria to distinguish MT1 from spondylitis, but with overall low to moderate diagnostic accuracy. For example, in a recent study, quantifications of the edema area within affected vertebral bodies yielded a sensitivity of approximately 80% for identifying MT1 lesions at a cut-off value of ≤55%, and were thus unlikely to exclude underlying infection with certainty [21]. Indistinct subchondral edema zones on T1- and T2-weighted MR images, generally suggestive of infectious rather than degenerative etiology, were shown to occur in both MT1 lesions and acute infectious spondylitis, allowing for a differentiation with an only low sensitivity and specificity of 64% and 68%, respectively [22]. Other frequently observed morphological alterations, such as significantly increased T2 signal in the adjacent intervertebral disc, have shown to be very specific for diagnosing spondylodiscitis but offer limited sensitivity because they may occur late in the course or even not at all despite clinically manifesting spondylitis [23]. In order to overcome the qualitative nature of imaging diagnosis, recent advances in MRI pulse sequence development have introduced quantitative tools for assessment of the vertebral bone marrow matrix. Parametric imaging biomarkers, which may reflect certain aspects of the pathophysiology and tissue microenvironment, could potentially contribute to appropriate diagnosis in addition to established clinical and imaging parameters. 

In our study, relative PDFF was quantitatively assessed by chemical-shift encoding-based water-fat MRI, thereby providing a technique to inversely measure the extent of edema within the bone marrow: bone marrow is one of the few tissues in the human body where both water and fat can be present in almost equal amounts [24]. In healthy subjects, vertebral bone marrow adipose tissue constitutes a major component of the cellular compartment and can vary, on average, between 27.2% and 50.5% depending on age and gender [25]. An increase in interstitial fluid within the marrow cavity leads to a relative decrease in estimated vertebral fat content, and vice versa, which can be quantified using PDFF. With a mean PDFF of 35.29% for MT1 degeneration and 4.28% for spondylitis, the PDFF showed statistically significant differences in our cohort, thus illustrating what has been learned from the anecdotal evidence: the more edema, the higher the likelihood of infection. Compared with healthy bone marrow (mean PDFF of 51.56%), the PDFF of both MT1 degeneration and spondylitis was also significantly decreased. Our results are therefore largely consistent with and quantitatively extend those of previous reports on the extent of edema in MT1 and spondylitis lesions. In light of the high diagnostic accuracy of 98.1% in our study compared with previous investigations, the importance of studying the bone marrow in more detail using quantitative imaging techniques becomes evident. We observed one false positive finding in a patient with radiological suspicion of infection later disproved clinically who had a corresponding vertebral mean PDFF of 1.9%, which was probably due to extensive edema in the affected vertebral bodies. This patient was diagnosed with an additional inflammatory abdominal aortic aneurysm showing retroperitoneal fibrosis with paravertebral involvement on the lumbar level. Consistent with the currently suspected inflammatory pathogenesis of EO [3], it can be suggested that the persistent proinflammatory condition in the adjacent soft tissue has led to an aggravation of MT1 endplate-associated edema. Interestingly, the remaining 3 of 4 patients who had an imaging suspicion of spondylitis based on conventional MRI (later disproved clinically) were correctly identified as having MT1 degenerative changes using PDFF. 

Alongside conventional MRI, diffusion weighted imaging (DWI) has been successfully applied to differentiate between EO and infectious bone marrow lesions. Diffusivity is reduced in tissue with high cellularity, e.g., in bone marrow densely infiltrated by inflammatory cells, due to a reduction of the free fluid component within the interstitial space. Conversely, in MT1 degenerative changes, the free water content is increased in the depleted bone marrow space, with a consequent increase of the measured apparent diffusion coefficient (ADC) value [26]. Previous studies have provided evidence for the usefulness of qualitative DWI-based spondylitis assessment, showing that a significant increase in signal intensity on DW images is highly suggestive of infection [27]. Particularly, a specific morphological pattern of band-like regions of restricted diffusion affecting two contiguous vertebral bodies (referred to as the “claw sign”) was highly predictive and accurately identified MT1 degenerative lesions in 97–100% of cases [23]. It has also been shown that quantitative DWI analysis can aid discrimination between MT1 and infectious spondylitis using ADC cut-off values in the range from ≤0.79 to ≤1.31 × 10^−6^ mm^2^/s for infection [28]. In general, however, there are certain technical limitations to the use of DWI for bone marrow assessment: first, most DWI techniques are not standardized across imaging sites, making it difficult to directly compare the observed results [29]. Second, DWI is prone to susceptibility artefacts induced at tissue boundaries especially in the trabecular bony matrix and at high field strengths [30]. Third, the large variation of fat amount in vertebral bone marrow may lead to misidentification of fat signal as arising from water, potentially leading to significant quantification errors of the ADC [31]. Fourth, its lowered sensitivity for sclerotic bone marrow may lead to false negative findings [32]. The complex-based water-fat MRI technique with multiple echoes, such as the modified Dixon method, has been shown to be able to largely minimize the effects of confounding factors in the determination of vertebral bone marrow fat content: T1 distortion is reduced by using low flip angle excitation, fat measurement is highly accurate by including a multi-peak spectral model with implementation of multiple lipid components, and T2* signal decay due to microscopic magnetic field inhomogeneity effects in trabecular bone is widely corrected computationally during the post-processing stage [9]. Thus, numerous in vitro and in vivo studies using either water-fat phantoms [33,34] or volunteer cohorts [35] have demonstrated that PDFF yields excellent precision across field strengths and vendor-based reconstruction methods.

## 5. Limitations

We acknowledge several limitations of this study. The retrospective nature generally limits the conclusion to be drawn since the repeatability of the obtained clinical and imaging data cannot be determined. Bioptic verification was not available in MT1 patients, which were mostly diagnosed based upon clinical features, negative blood culture results, and imaging findings. This, however, reflects the situation in clinical routine as well as ethical considerations, since patients with apparently degenerative endplate changes usually do not undergo biopsy. The sample size of our cohort was relatively small because all patients had to undergo a standardized 3D-spoiled six-echo CSE-based water-fat MRI, which is not yet routinely used to evaluate lower back pain. However, the statistical significance and the high predictive values in this evaluation should favor efficacy of PDFF measurements in a clinical setting. Image acquisition at different field strengths may have influenced the precision of the obtained quantitative imaging data; however, dedicated field strengths comparisons demonstrated statistically insignificant and only marginal average differences in PDFF and PDFFratio measurements among the 1.5T and 3T scanners, which in most cases are likely to be clinically irrelevant for the differentiation of MT1 and infectious lesions.

## 6. Conclusions

This study introduces PDFF derived from CSE-based water-fat MRI as a quantitative imaging parameter that is highly accurate in distinguishing spinal MT1 degenerative changes from infectious spondylitis. The PDFF may usefully supplement classic MR imaging features and can potentially increase accuracy and confidence in the differential diagnosis of MT1 degenerative changes versus spondylitis of the spine. Owing to the excellent diagnostic accuracy and high NPVs of PDFF, implementation of quantitative water-fat spine MRI in clinical practice may potentially reduce cost by eliminating concern for infection in symptomatic patients manifesting with MT1 changes, which might otherwise provoke bone biopsy or surgical intervention. 

## Figures and Tables

**Table 1:** Group differences between MT1 erosive endplate degeneration and infectious spondylitis.

Variable		Modic Type 1 Degenerative Changes		Infectious Spondylitis		*p*
		Mean	±SD	Mean	±SD	
PDFF	%	35.29	17.15	4.28	3.12	<0.001 *
Normal vertebral body PDFF	%	57.41	13.76	43.32	18.58	0.003 *
PDFF_ratio_		0.67	0.37	0.093	0.059	<0.001 *
ROI size	mm^2^	179	109	283	159	0.003 *

*, at *p* < 0.05 statistically significant. PDFF, proton density fat fraction; ROI, region of interest; SD, standard deviation.

**Table 2:** Subgroup PDFF values.

Variable	Group	N	Mean	±SD	SE
PDFF	Clinical and imaging suspicion of infection (group 1)	22	4.28	3.12	0.66
	Modic type 1 degenerative changes (group 2)	27	37.71	16.43	3.16
	Imaging suspicion of infection without clinical evidence (group 3)	4	18.96	13.84	6.92
Normal vertebral body PDFF	Clinical and imaging suspicion of infection (group 1)	22	43.32	18.58	3.96
	Modic type 1 degenerative changes (group 2)	27	59.37	12.86	2.47
	Imaging suspicion of infection without clinical evidence (group 3)	4	44.15	13.82	6.91
PDFFratio	Clinical and imaging suspicion of infection (group 1)	22	0.09	0.06	0.01
	Modic type 1 degenerative changes (group 2)	27	0.70	0.37	0.07
	Imaging suspicion of infection without clinical evidence (group 3)	4	0.47	0.29	0.15

PDFF given in units of %. SD, standard deviation; SE, standard error.

**Table 3:** Subgroup comparisons of quantitative PDFF imaging parameters.

Variable	Group	Comparison Group	Mean Difference	SE	*p*
PDFF	Clinical and imaging suspicion of infection (group 1)	Modic type 1 degenerative changes (group 2)	−33.42	3.58	<0.001 *
		Imaging suspicion of infection without clinical evidence (group 3)	−14.67	6.79	0.106
	Modic type 1 degenerative changes (group 2)	Clinical and imaging suspicion of infection (group 1)	33.42	3.58	<0.001 *
		Imaging suspicion of infection without clinical evidence (group 3)	18.75	6.69	0.022 *
	Imaging suspicion of infection without clinical evidence (group 3)	Clinical and imaging suspicion of infection (group 1)	14.67	6.79	0.106
		Modic type 1 degenerative changes (group 2)	−18.75	6.69	0.022 *
Normal vertebral body PDFF	Clinical and imaging suspicion of infection (group 1)	Modic type 1 degenerative changes (group 2)	−16.05	4.47	0.002 *
		Imaging suspicion of infection without clinical evidence (group 3)	−0.83	8.46	1
	Modic type 1 degenerative changes (group 2)	Clinical and imaging suspicion of infection (group 1)	16.06	4.47	0.002 *
		Imaging suspicion of infection without clinical evidence (group 3)	15.22	8.34	0.222
	Imaging suspicion of infection without clinical evidence (group 3)	Clinical and imaging suspicion of infection (group 1)	0.83	8.46	1
		Modic type 1 degenerative changes (group 2)	−15.22	8.34	0.222
PDFFratio	Clinical and imaging suspicion of infection (group 1)	Modic type 1 degenerative changes (group 2)	−0.61	0.08	<0.001 *
		Imaging suspicion of infection without clinical evidence (group 3)	−0.38	0.15	0.051
	Modic type 1 degenerative changes (group 2)	Clinical and imaging suspicion of infection (group 1)	0.61	0.08	<0.001 *
		Imaging suspicion of infection without clinical evidence (group 3)	0.23	0.15	0.41
	Imaging suspicion of infection without clinical evidence (group 3)	Clinical and imaging suspicion of infection (group 1)	0.38	0.15	0.051
		Modic type 1 degenerative changes (group 2)	−0.23	0.15	0.41

*, at *p* < 0.05 statistically significant. PDFF given in units of %. SE, standard error.

**Table 4:** Diagnostic performance of PDFF and PDFFratio for differentiating Modic type 1 degenerative lesions from infectious spondylitis.

Parameter	AUC	SE	*p*	CI1	CI2	Cut-Off	Sen	Spec	Acc
PDFF	0.977	0.023	<0.001 *	0.931	1	12.9	1	0.97	0.98
PDFFratio	0.971	0.29	<0.001 *	0.914	1	0.27	1	0.97	0.98

*, at *p* < 0.05 statistically significant. Cut-off points are given in units of % for PDFF. AUC area under the curve, SE standard error, *p* significance level, CI 95% confidence interval, Sen sensitivity (true positive rate), Spec specificity (true negative rate), Acc accuracy (rate of correctly identified cases).

## Data Availability

The data presented in this study are not publicly available due to privacy restrictions. The data are, however, available on reasonable request from the corresponding author.
